# Dislocations of the acromioclavicular and sternoclavicular joint in children and adolescents: A retrospective clinical study and big data analysis of routine data

**DOI:** 10.1371/journal.pone.0244209

**Published:** 2020-12-28

**Authors:** Ralf Kraus, Joern Zwingmann, Manfred Jablonski, M. Sinan Bakir

**Affiliations:** 1 Department of Trauma Surgery and Orthopedics, Klinikum Bad Hersfeld GmbH, Bad Hersfeld, Germany; 2 Section of Pediatric Traumatology (Sektion Kindertraumatologie, SKT) of the German Trauma Society (Deutsche Gesellschaft für Unfallchirurgie, DGU), Berlin, Germany; 3 Department of Trauma Surgery and Orthopedics, St. Elisabethen Klinikum, Ravensburg, Germany; 4 Department of Pediatric Surgery and Urology, Kinderkrankenhaus Auf der Bult, Hannover, Germany; 5 Department of Trauma and Reconstructive Surgery and Rehabilitative Medicine, Medical University Greifswald, Greifswald, Germany; 6 Department of Trauma Surgery and Orthopedics, BG Hospital Unfallkrankenhaus Berlin gGmbH, Berlin, Germany; University Hospital Zurich, SWITZERLAND

## Abstract

**Background:**

Dislocations of the sternoclavicular joint (anterior/posterior) and acromioclavicular joint (SCJ and ACJ, respectively) are rare injuries in childhood/adolescence, each having its own special characteristics. In posterior SCJ dislocation, the concomitant injuries in the upper mediastinum are most important complication, while in anterior SCJ dislocation there is a risk of permanent or recurrent instability.

**Methods:**

In a retrospective analysis from seven pediatric trauma centers under the leadership of the Section of Pediatric Traumatology of the German Trauma Society, children (<18 years) were analyzed with focus on age, gender, trauma mechanism, diagnostics, treatment strategy and follow-up results. Additional epidemiological big data analysis from routine data was done.

**Results:**

In total 24 cases with an average age of 14.4 years (23 boys, 1 girl) could be evaluated (7x ACJ dislocation type ≥ Rockwood III; 17x SCJ dislocation type Allman III, including 12 posterior). All ACJ dislocations were treated surgically. Postoperative immobilization lasted 3–6 weeks, after which a movement limit of 90 degrees was recommended until implant removal. Patients with SCJ dislocation were posterior dislocations in 75%, and 15 of 17 were treated surgically. One patient had a tendency toward sub-dislocation and another had a relapse. Conservatively treated injuries healed without complications. Compared to adults, SCJ injuries were equally rarely found in children (< 1% of clavicle-associated injuries), while pediatric ACJ dislocations were significantly less frequent (p<0.001).

**Conclusions:**

In cases of SCJ dislocations, our cohort analysis confirmed both the heterogeneous spectrum of the treatment strategies in addition to the problems/complications based on previous literature. The indication for the operative or conservative approach and for the specific method is not standardized. In order to be able to create evidence-based standards, a prospective, multicenter-study with a sufficiently long follow-up time would be necessary due to the rarity of these injuries in children. The rarity was emphasized by our routine data analysis.

## Introduction

The sternoclavicular and the acromioclavicular joints (SCJ and ACJ, respectively) are the only joints in the general sense that are found in the connection between the trunk and upper extremities. Both joints are involved in all movements of the entire arm. The SCJ forms the tip, while the ACJ forms the basis of a conical movement sector. Both joints are protected by strong ligaments (medial: sternoclavicular, interclavicular and costoclavicular ligaments, lateral: acromioclavicular and coracoclavicular ligaments).

Accordingly, dislocations of these two joints are rare injuries in childhood and adolescence. In particular, in childhood periarticular metaphyseal clavicle fractures or epiphysiolysis of the clavicle seem to be significantly more common [1, 2]. The literature concerning these injuries is sparse. Systematic studies of larger cohorts are missing; many publications only refer to individual accounts. An overview of non-traumatic differential diagnoses can be found in Robinson et al. [3]. A differential diagnostic algorithm is necessary due to the rather rare pattern of pediatric SCJ and ACJ injuries. The imaging diagnostics complement the clinical examination. Native X-rays (for ACJ, often insufficient in case of SCJ), ultrasound, and computed tomography (CT) scans are used, while use of magnetic resonance imaging (MRI) is rather uncommon [4–6].

**Fig 1:**
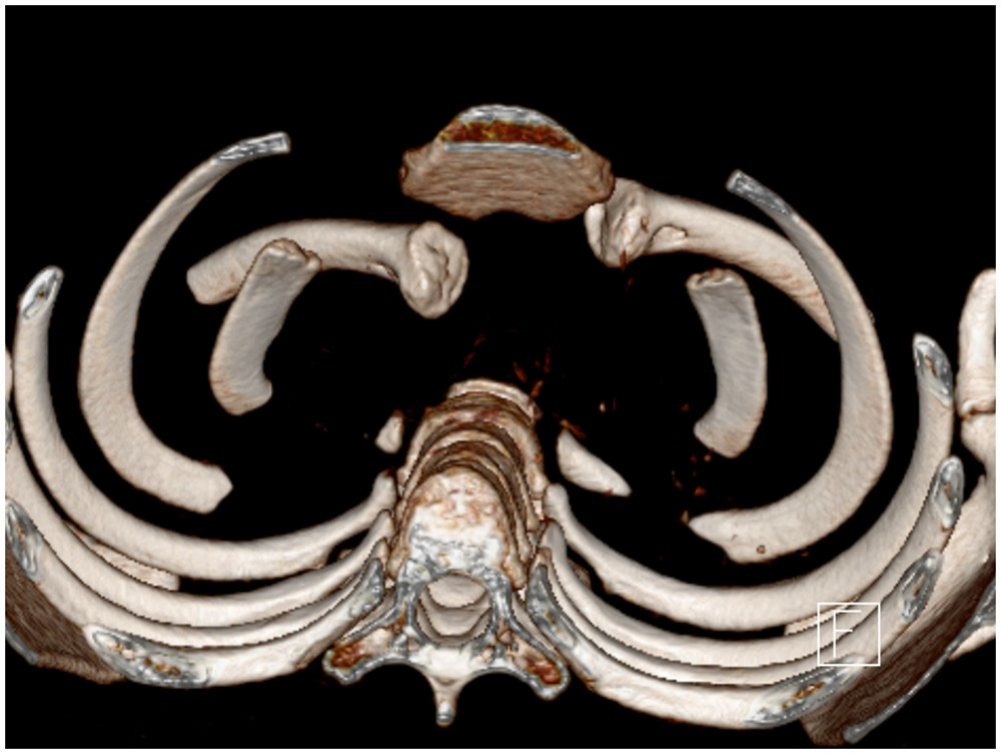
Three-dimensional computed tomography of a posterior sternoclavicular joint (SCJ) dislocation (10-year-old boy, road traffic accident).

Three regularly recurring injury groups can be defined consisting of ACJ dislocation and both anterior and posterior SCJ dislocations, each having its own special characteristics. In posterior SCJ dislocation, the concomitant injuries in the upper mediastinum play an important role in the literature, but previous literature is not related to the situation in children and adolescents (Fig 1). Concomitant injuries are related to arterial and venous vascular compressions, tracheal and esophageal injuries, and space-consuming mediastinal hematomas [4, 7, 8]. The major complication of anterior SCJ dislocation is a permanent or recurrent instability [5, 9, 10]. With regard to the treatment of ACJ injury, previous recommendations offer the equality of operative and conservative therapy similar to that in adults depending on the age of the children [11, 12].

As the primary objective of this study, we present the importance of pediatric SCJ and ACJ dislocations relative to their incidence. Therefore, we investigated concomitant injuries and applied treatment strategies based on both clinical data and current epidemiological data. Our secondary goal was to answer the question as to whether there is a difference in adults and children concerning these clavicular joint injuries.

## Methods

In a retrospective cohort analysis in association with the scientific working group of the Section of Pediatric Traumatology of the German Trauma Society (Deutsche Gesellschaft für Unfallchirurgie, DGU), children and adolescents up to the age of 18 years with isolated ACJ and SCJ dislocations from November 2013 to December 2018 were identified. Children with further injuries related to the clavicle, such as periarticular fractures were excluded from the clinical investigation.

Age, gender, trauma mechanism, and additional concomitant injuries were recorded. SCJ dislocations have also been categorized based on the direction of dislocation. In addition, conservative and surgical treatments were analyzed in detail as the surgical fixation method, postoperative treatment, and time of implant removal if necessary. As part of the follow-up examinations, the range of movement and potential instabilities were assessed.

Epidemiological data were provided by the German Federal Statistical Office and include all inpatients from all German hospitals according to diagnosis-related groups (DRGs) in the scope of § 1 of the German Hospital Finance Law (KHEntgG) [13]. These anonymized routine data are based on the 10th revision of the International Statistical Classification of Diseases and Related Health Problems (ICD-10 codes) [14]. In particular, the ICD-10 codes S43.1 and S43.2 (acromioclavicular and sternoclavicular joint dislocations) were evaluated for a period of three years. We retrospectively performed a big data analysis concerning the incidence, age, and sex distribution from 2012 to 2014 and compared them to data from adults > 20 years old. The German Federal Statistical Office divided the ICD-10-related age data basically into 5-year age ranges. Since the interval between 15 and 20 years includes pediatric and adult patients, this range was considered separately from both groups to avoid confounding factors.

Informed consent was obtained from all individual participants included in the study and their legal guardians, including participation and publication of the results/potential identifying information. The study was performed in accordance with the ethical standards laid down in the 1964 Declaration of Helsinki and was registered with the German Clinical Trials Register (DRKS; DRKS00017018) after approval by the local ethics committee (Medical University Greifswald, BB 007/19).

The analysis of the clinical data was performed exclusively using descriptive methods. Due to the small sample size and inhomogeneous cohort in relation to the selected treatment methods and epidemiological aspects, formal statistical testing was avoided since even major (and clinically relevant) differences would not be statistically significant. Therefore, the calculation of significance, standard deviations, and confidence intervals appeared as not expedient. The statistical epidemiologic analysis of the routine data was performed using SPSS software (IBM SPSS Statistics for Windows, Version 26.0. Armonk, NY: IBM Corp.) and associations were tested by Pearson’s chi-squared test. Fisher’s exact test with an alpha level of 0.05 was used in the case of expected cell values less than n = 5. Due to the explorative nature of the analysis, no alpha adjustment for multiple testing was conducted.

## Results

A total of 24 cases from seven trauma centers were analyzed for the study, which met the admission criteria for pure luxation of the SCJ or ACJ. The average age was 14.4 years (range 10–17 years, median 14 years). The male gender far outweighed females at 23:1.

**Fig 2:**
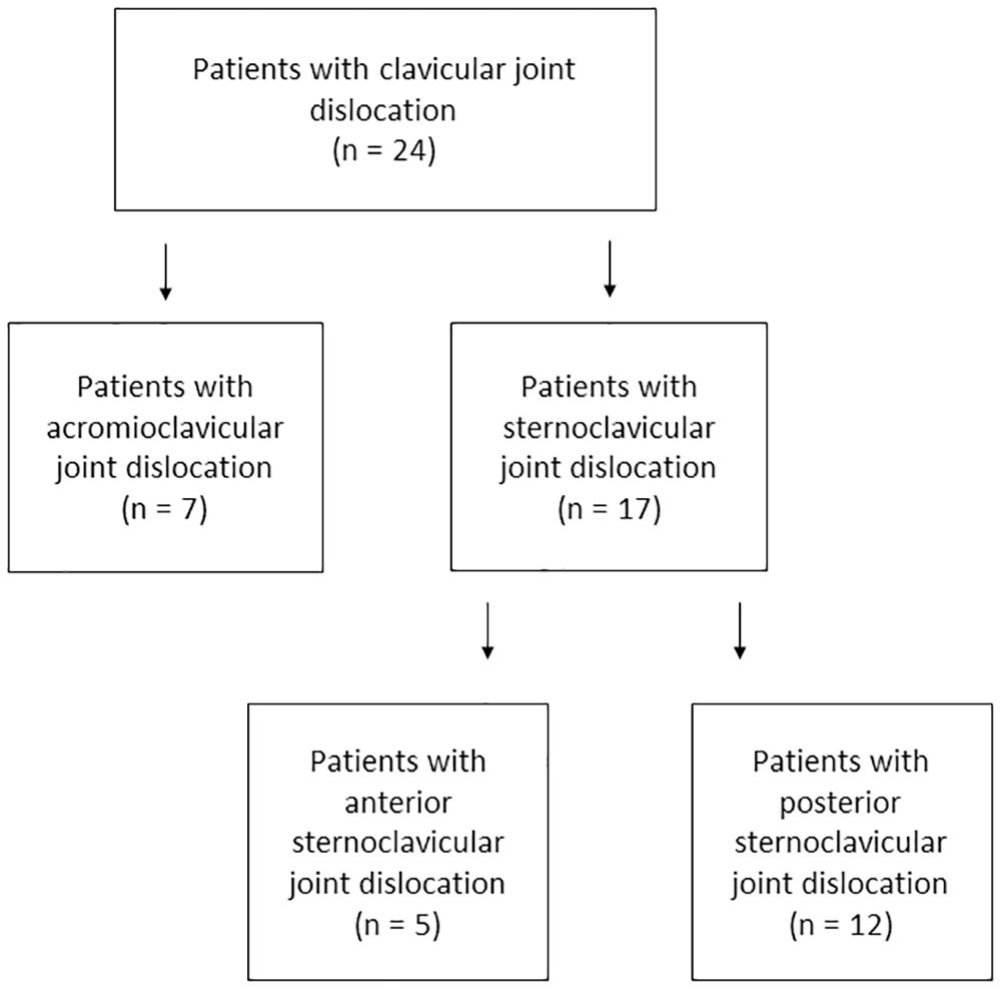
Distribution of clavicular joint dislocations. The clinical cases from seven participating trauma centers were divided into acromioclavicular joint dislocation or (anterior/posterior) sternoclavicular joint dislocation.

Among the 24 injuries, seven ACJ dislocations and 17 SCJ dislocations were found (Fig 2). All AC joint dislocations were unstable injuries, referring to type Tossy III / Rockwood III–V (Table 1). The type of the SCJ dislocations was a type Allman III in all cases, in the anterior direction in five cases and in the posterior direction in 12 cases (Table 2).

**Table 1:** Personal data, trauma mechanism, type of injury referring to Rockwood classification and surgical methods for seven acromioclavicular joint (ACJ) dislocations.

Nr.	Gender	Age	Trauma mechanism	Rockwood classification	Method of surgery	Time of implant removal
1	Male	13	Soccer	III	Tension flange	6 weeks
2	Male	16	Fall	IV	Hook plate	6 months
3	Male	17	Soccer	III	Hook plate	3 months
4	Male	11	Bicycle	III	K wire	6 weeks
5	Male	16	Bicycle vs. Car	V	Hook plate	6 months
6	Male	14	Sports	III	Hook plate	3 months
7	Male	15	Soccer	III	Tight Rope^®^	-

**Table 2:** Personal data, trauma mechanism, type of injury referring to Allman classification and surgical methods for 17 sternoclavicular joint (SCJ) dislocations.

Nr.	Gender	Age	Trauma mechanism	Allman classification	Direction of dislocation	Primary treatment
1	Male	13	Playing	III	posterior	PDS Banding
2	Male	13	Skiing	III	anterior	Suture-anchor-system
3	Male	15	Sports	III	anterior	Suture-anchor-system
4	Male	16	Fall	III	anterior	PDS Banding + gracilis plastic
5	Male	15	Bicycle	III	posterior	PDS Banding
6	Male	15	Fall	III	anterior	Suture-anchor-system
7	Male	12	Skiing	III	posterior	PDS Banding
8	Male	10	Pedestrian versus Car	III	posterior	PDS Banding
9	Male	12	Skiing	III	posterior	Closed reduction
10	Male	15	Bicycle versus Car	III	posterior	PDS Banding
11	Male	12	Fall	III	posterior	Suture cerclage
12	Male	16	Bicycle versus Car	III	posterior	K wire cerclage
13	Female	15	Swimming	III	posterior	Suture cerclage
14	Male	14	Soccer	III	posterior	Closed reduction
15	Male	13	Gymnastics	III	anterior	PDS Banding
16	Male	10	Fall	III	posterior	PDS Banding
17	Male	15	Bicycle	III	posterior	K wire cerclage

### Trauma mechanism

Three patients injured themselves while playing (two falls from the climbing frame). Sports accidents occurred in 14 cases. General sports and soccer (n = 5 in both cases) were mentioned most frequently. In addition, three skiing accidents were reported. The only injured girl suffered a water sports accident. Out of seven traffic accidents, in two cases, bicycle accidents without external influence occurred, and two events involved pedestrians and cars and three events bicycles and cars. In relation to the severity of the injury, one poly-traumatized and two multiple-injured patients in addition to 21 mono-injuries to the clavicle related shoulder girdle were found.

### Therapeutic strategy

Only three patients (all SCJ dislocations) were treated conservatively, primarily by closed reduction. In one of these cases, an open reduction had to be performed secondarily. All other cases, including all ACJ dislocations, were primarily treated with surgery (n = 21).

**Fig 3:**
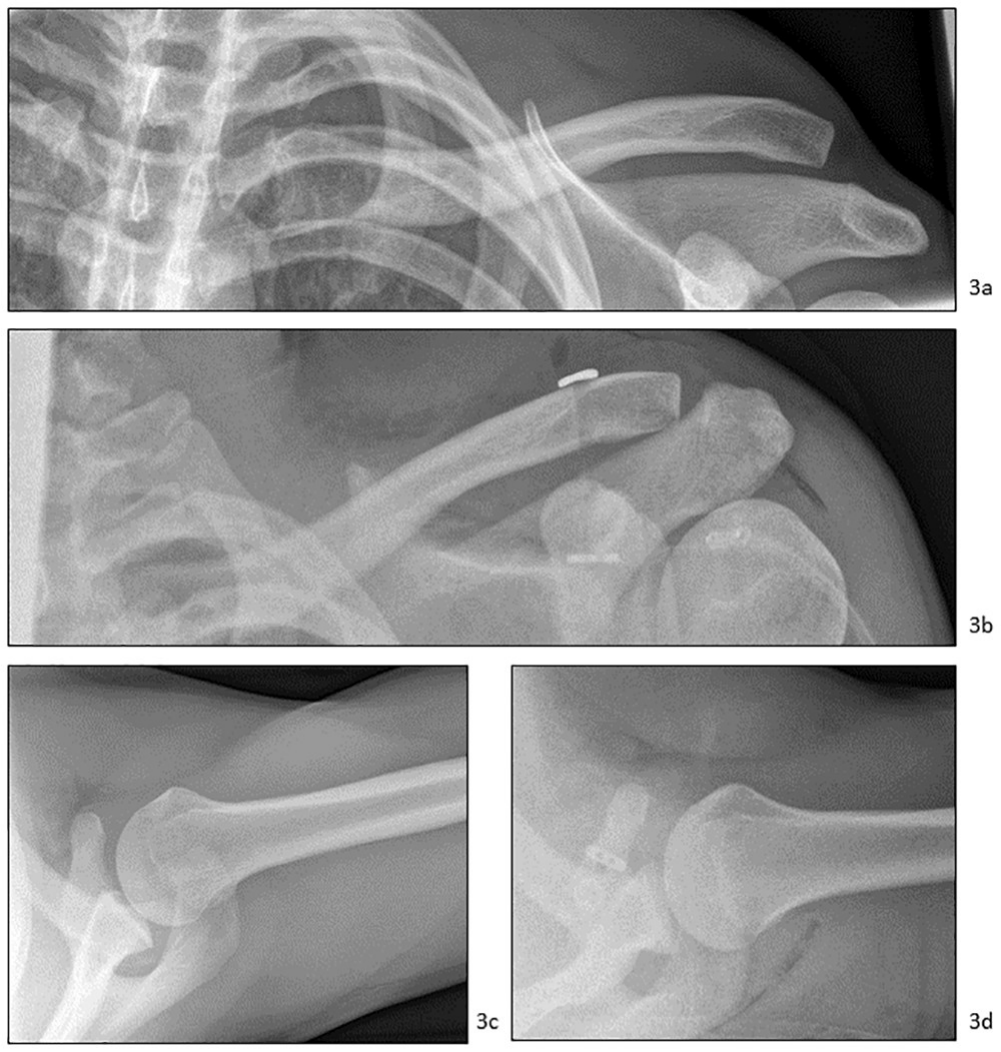
Acromioclavicular joint dislocation treated with Tight Rope^®^ stabilization. Preoperative and postoperative x-rays of an acromioclavicular joint (ACJ) dislocation type Rockwood III with horizontal instability treated with Tight Rope^®^ stabilization (15-year-old boy, fall while playing soccer). a) Preoperative anterior-posterior view. b) Postoperative anterior-posterior view. c) Preoperative axial view. d) Postoperative axial view.

**Fig 4:**
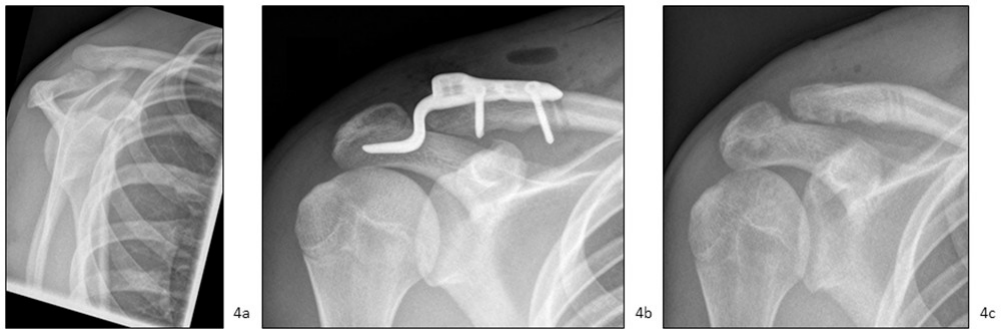
Acromioclavicular joint dislocation treated with hook plate. Preoperative and postoperative x-rays of an acromioclavicular joint (ACJ) dislocation type Rockwood III (horizontal unstable) with surgical treatment using a hook plate (16-year-old boy, fell while playing soccer, implant removal after nine weeks). a) Preoperative x-rays. b) Postoperative x-rays. c) X-rays after implant removal.

In one case, the ACJ dislocations were treated with a Tight Rope^®^ (Fig 3), in two cases with K wires or a tension flange, and four cases with a hook plate (Fig 4). The immobilization period was between three and six weeks. The implants were removed after six weeks in cases of tension flange and three to six months in cases of hook plates (Table 1). The range of motion in the period between the end of immobilization and the implant removal was limited in all cases to 90 degrees of anteversion and abduction. In the case of the Tight Rope^®^, abduction was limited to 90 degrees over six weeks.

The SCJ dislocations were fixed in 10 cases with suture cerclages (mostly PDS banding), three times with suture-anchor-systems and two times with K wire cerclages. Suture-anchor-systems were only used for anterior dislocations. The K wire cerclages were removed after six weeks in both cases (Table 2). Postoperative immobilization was three to six weeks with an average of 4.4 weeks. Limited anteversion and abduction was recommended for further three to twelve weeks at 70 to 90 degrees. In the case of the secondary surgical intervention after four weeks, a gracilis plastic was used in addition to a suture-anchor-system. As the only one of the patients treated surgically, he later suffered a relapse of primary anterior SCJ dislocation.

The follow-up period lasted up to three years (on average median of 13 months). One relapse and one stress-dependent tendency to a sub-dislocation occurred. Both concerned SCJ dislocations, each after anterior and posterior dislocation, respectively. No significant constraints on range of movement at the last time of the follow-up examination each were found.

### Epidemiological big data analysis

For the epidemiological analysis, we examined children under 15 years (n = 4229 patients with clavicular injuries), adolescents from 15 to 20 years (n = 7910) and adults older than 20 years (n = 101,864). The SCJ injuries were equally rare in children compared to adults (p = 0.874), each with considerably < 1% of all clavicle-associated injuries, while the proportion was significantly higher (p < 0.001–0.007) in the adolescent group on a low level (Table 3). In contrast, the proportion of ACJ dislocations was clearly significantly less frequent in the pediatric group (p < 0.001) than in the adolescent and adult group with increasing amount proportional to age.

**Table 3:** Distribution of clavicular injuries according to age.

	SCJD	MCF	MICF	LCF	ACJD	Total
**<15 years**						
total	0.5%	17.5%	57.7%	20.7%	3.4%	100.0%
male	0.5%	15.6%	56.3%	23.7%	3.9%	100.0%
female	0.6%	22.0%	60.9%	14.1%	2.4%	100.0%
**15–20 years**						
total	1.0%	14.7%	57.0%	13.2%	14.1%	100.0%
male	1.1%	14.3%	56.4%	13.0%	15.2%	100.0%
female	0.8%	16.4%	59.3%	14.1%	9.3%	100.0%
**>20 years**						
total	0.6%	11.5%	33.6%	26.5%	27.9%	100.0%
male	0.5%	10.8%	34.0%	21.7%	33.1%	100.0%
female	0.7%	13.3%	32.6%	39.5%	13.9%	100.0%

Comparison of children < 15 years versus adolescents 15–20 years versus adults > 20 years. The numbers are presented as the percentage of all clavicle-related injuries. SCJD = sternoclavicular joint dislocation; MCF = medial clavicle fracture; MICF = midshaft clavicle fracture; LCF = lateral clavicle fracture; ACJD = acromioclavicular joint dislocation.

While the overall gender ratio differed only slightly (children’s group boys / girls: 69.1% / 30.9%; adult group male / female: 73.2% / 26.8%), a significant (p < 0.001) increase in the proportion in favor of ACJ dislocations of male adult patients was found (Table 3). This tendency in favor of male ACJ dislocations is also evident in the adolescent group, albeit to a lesser extent (p < 0.001). However, the overall gender ratio is most diverse in this group (male / female: 82.5% / 17.5%).

The concomitant injuries in pediatric CJI were particularly evident in the area of the head, thorax and upper extremity (Table 4). There was a significant difference in the thoracic concomitant injuries with predominance in SCJ injuries (p = 0.002) and in the shoulder/upper extremity, which were significantly more common in ACJ dislocations (p < 0.001).

**Table 4:** Epidemiological analysis of concomitant injuries of children < 15 years.

	SCJD	ACJD
**Head**	4.3%	20.0%
**Neck**	13.0%	2.8%
**Thorax**	26.1%*	4.1%*
**Abdomen, Spine, Pelvis**	4.3%	6.2%
**Shoulder, Upper Arm**	13.0%*	55.2%*
**Elbow, Forearm**	0.0%	3.4%
**Wrist, Hand**	0.0%	0.0%
**Hip, Thigh**	0.0%	0.7%
**Knee, Shank**	4.3%	1.4%
**Ankle, Foot**	0.0%	1.4%

Comparison of both entities of clavicular joint injuries. The numbers are presented as the percentage in each relation to the total number of the respective injury. SCJD = sternoclavicular joint dislocation; ACJD = acromioclavicular joint dislocation;

* = significant difference p ≤ 0.05.

## Discussion

One of the foundations of this study is the assumption that when considering SCJ and ACJ injuries in childhood and adolescence, a distinction should be made between dislocated fractures (metaphyseal or epiphysiolysis) and actual dislocations as also pointed out in the literature [12, 15]. While there is always a certain capability of remodeling in case of joint-related fractures due to growth-related potential for corrections, no such possibility in the case of actual SCJ or ACJ dislocations exists. In addition, the surgical methods differ distinctly between the options for surgical fixation for joint-related fractures in contrast to the different operative procedures in joint dislocations.

Real dislocations of the SCJ and the ACJ in childhood and adolescence are very rare injuries [7, 8, 16, 17]. Therefore, generally valid therapy recommendations are missing, both in terms of the indication for the conservative versus operative therapy, as well as the particular surgical reconstruction and fixation techniques [10]. Dorsal SCJ dislocations are mostly described in the literature because of life-threatening mediastinal concomitant injuries, while anterior SCJ dislocation is mentioned more often because of remaining chronic instabilities [4, 9, 18]. The problem of chronic instability also applies to untreated ACJ dislocation as the major posttraumatic complication [11].

Concerning the age distribution in our clinical analysis, it can be seen that these injuries did actually not occur in our patient cohort under the age of ten years. This finding coincides with findings in the literature [12]. We assume that children with a similar injury mechanism are more likely to fracture the clavicle. Most of the patients were adolescents who sustained injuries from low-energy trauma. The proportion of those undergoing high-energy trauma, which plays a major role in adults with SCJ injuries, particularly caused by traffic accidents, was low in our patient population [1, 19, 20]. This finding could be a reason why the proportion of mono-injuries in our patient population was higher than in adults. The latter show a greater proportion of concomitant injuries, particularly on the upper extremity and also as thoracic injuries [21].

### SCJ dislocation

While in the case of dorsal SCJ dislocation, severe mediastinal and thoracic concomitant injuries, such as arterial compression syndromes, esophageal, and lung injuries are described in the literature, only one lung contusion was found among the analyzed cohort as an additional, anatomically close localized injury. Nevertheless, prompt reduction of the dislocation took place in all cases of patient population since all of them suffered high-grade and dislocated Allman III dislocations. The literature also points out the need for immediate reduction [8, 22, 23].

However, while some authors believe that closed reduction alone is sufficient and promising in the long term, others favor immediate surgical stabilization of the joint [5, 7, 24, 25]. In this context, Tepolt et al. report that six of the eight primarily closed dorsal SCJ dislocations required secondary surgical stabilization [26]. In our own patient population, 10 of 12 dorsal SCJ dislocations were primarily openly reduced and stabilized. Here, eight suture cerclages and two K wire cerclages were used, which are similar approaches as described by Cheng and Waters et al. [7, 27]. For all posterior dislocations, no closed reduction was possible so that an open reduction was carried out to avoid compression-related complications and complaints analogous to the literature, which was then performed together with SCJ stabilization [18]. Interestingly, less than 1/3 of the patients (5 of 17) in our pediatric collective had anterior dislocations, while the ratio in adults being up to 20:1 [28]. It can only be assumed whether the mostly expected trauma mechanism with a compression of the shoulder girdle from posterolateral was present, thus causing a posterior dislocation in the SCJ [29]. Mainly sports and traffic accidents were responsible in our cohort, which are similar to the adult injury mechanisms known to date [30]. Whether the different connective tissue with laxer capsular ligament structures in the area of the SCJ in the child’s skeleton favor this is our hypothesis that should be investigated in biomechanical studies. On the follow-up examinations after up to 36 months, all of our patients, both the surgically stabilized and the conservatively closed reduced patients showed normal findings in terms of range of motion and stability. We did not see any growth disturbances in our patient group, which could have been suspected due to the potentially compromising osteosynthesis material and the open epiphyseal plates.

Strict criteria for the indication of a surgical procedure as set out by Allman for adults could not be identified as also shown in the current corresponding literature [31].

The problem with anterior SCJ dislocation is less in possible concomitant injuries than in the development of chronic instability [9, 32]. This can arise after primary conservative therapy as well as after primary surgical care. To avoid this, more elaborate procedures are described in the literature, such as the combination of cerclages and reconstruction of capsule/muscles and tendon plastic, which are also used for the secondary treatment of remaining instabilities [10, 32, 33]. Above all, Thut et al. describe the experience in which the use of tendon grafts promises more lasting stability than the reconstruction with local tissue alone [33]. In the event of secondary instability, the partly resection of the medial clavicle is also proposed as the ultima ratio [5, 9]. Although this is mentioned as a last resort with equivalent results for joint reconstruction even among adolescents with chronic instability, we emphasize that this must be an absolute exception in our opinion, especially in the immature skeleton, and must be very strictly indicated [9]. Four of the five cases were primarily treated with a PDS cerclage or suture-anchor-system. Periosteum and joint capsule were sutured in all cases. In one of these cases, a tendency to subluxation remained but did not require treatment. In the case of the adolescent who was initially treated conservatively for four weeks, treatment with PDS banding and additive gracilis plastic was performed due to clinical persistent instability. Unfortunately, this procedure ended in an unsatisfactory result with instability relapse. The heterogeneous treatment strategies coincide with the lack of therapy standards in the adult area for SCJ injuries [21].

### ACJ dislocation

Treatment of ACJ dislocation is still controversial in adults, especially in case of Rockwood III injuries [34, 35]. While many authors consider the stabilization of the joint to be urgently indicated in the case of these higher-grade injuries, others point to the good results after conservative treatment [35]. It is therefore not surprising that there are no treatment guidelines for the much rarer injuries in childhood and adolescence since these treatment recommendations are already inhomogeneous in adulthood [34]. In case of pediatric ACJ injuries, Meixner and Black et al. recommended conservative treatment of dislocations such as slightly displaced fractures, while Klein et al. favored surgical stabilization in the event of complete rupture of the lateral clavicular ligamentous apparatus [16, 36, 37]. Nenopoulos et al. found no functional difference between surgical and conservative treatment, but emphasized that the cosmetic outcome after surgery was better by avoiding the clavicle being above the actually anatomic level [11]. On the other hand, this analysis did not take into account the obligatory resulting scar formation after surgery, which could end in an even worse cosmetic result, especially in the case of keloid formation [11]. Eidmann et al. recommend a (surgical) treatment similar to adults for children above age thirteen, since they suffer from adult-type unstable ACJ dislocations, while a conservative treatment should produce good results in children below [12].

Different surgical methods are described. They range from reduction and transfixation with K wires to the implantation of tension flanges or hook plate. All seven patients in our own analysis were treated surgically. This is in line with the results of an online survey from German clinics, in which Rockwood III injuries are mainly treated surgically, which is the most frequent type in our population [38]. Since one of the injuries was a Rockwood IV and V each and all Rockwood III injuries were described as horizontal unstable, all injuries seemed to be regarded as unstable and therefore as an indication for surgery. In addition to cerclages and hook plates, indirect stabilization with Tight Rope^®^ was used. The latter has the advantage that it avoids the risk of additionally compromising the ACJ or any other nearby structures of the shoulder joint such as the subacromial space, but requires a larger and more demanding surgical effort. Due to the smaller joint in adolescents, the resulting mismatch between surgical arthroscopy instruments and space in the joint and the partial distribution and usage so far, arthroscopic procedures could be associated with an increase in complication rates. This finding should be weighed against the advantage that there is no need for implant removal in the case of Tight Rope^®^ stabilization. In our opinion, there is not enough experience so far. All of our patients achieved a free range of motion without recurrent dislocation relapse after the material removal so that all procedures used appear to be appropriate in the respective case.

A dislocation of the ACJ with an avulsion of the coracoid process, as described by Combalia et al. and Jetto et al., did not occur in our patient population [24, 39]. In this particular entity of an ACJ injury, it can be assumed that the coracoclavicular ligament remains uninjured, but both authors recommended surgical stabilization.

### Epidemiological big data analysis

Based on our epidemiological analysis of the routine data, the incidence of SCJ injuries is equally rare, as previously reported in the literature [40]. What was striking, however, was a significant difference in relation to the ACJ injury, which was hardly found in childhood and which, however, accounts for an increasing proportion in adolescence and a markedly large proportion in adulthood. We explain this difference by the infantile skeleton and the increased elasticity of the ligament structures in childhood, which make injuries in the ligamentous area of the ACJ less likely. Joint fractures or clavicular fractures with dislocation of the fragment cranially through the periosteal tube are more likely. Often these dislocated lateral clavicle fractures are erroneously diagnosed as having an ACJ lesion due to the rupture of the coracoclavicular ligament complex [12]. This coincides with the literature and with our clinical analysis in which ligamentous injuries are generally only to be expected in adolescence after the epiphyseal closure [2, 12, 41]. The low incidence in childhood also explains the low number of cases in our clinical retrospective study, since according to our epidemiological analysis, only 50 pediatric ACJ dislocations are treated inpatient per year in Germany. The gender difference only develops with increasing age, which coincides with experience in the literature and was there attributed to the missing role models and therefore the most frequent causes of accidents [40].

In contrast to our clinical cohort analysis, concomitant injuries of pediatric CJI mainly occurred in the head, thorax and upper limb regions in our epidemiological analysis, which corresponds to data from medial and lateral clavicular injuries in adults [21]. However, these showed the medial clavicular injuries with significantly more frequent concomitant injuries, whereby in our cohort the pediatric ACJ dislocations were significantly more often associated with concomitant injuries in the area of the shoulder/upper extremity [21]. This distribution can be explained due to the close anatomic distance, as can the more common thoracic concomitant injuries at SCJ dislocation [21].

There is a potential limitation in the interpretation of the results due to a selection bias: the cases are often searched for in a data query in the clinical information system using the ICD-10 code, which is, however, consistently coded and documented only for inpatients. This tends to document and record the patients who have suffered injuries with a higher degree of severity of the respective injury and were therefore treated in hospital [40]. As a result, these were mostly patients who needed surgery, which could explain the relatively high number of surgeries compared to the literature. In our collective, all injures were high-grade injuries ≥ Rockwood III ACJ dislocation / Allman III SCJ dislocations. Less complex cases with an uncomplicated course, which were treated on an outpatient and conservative basis, would therefore fall through the grid or would at least be underrepresented [42]. Unfortunately, this is a common bias in big data analysis [40].

The age interval between 15 and 20 years was considered a separate group for epidemiological comparison since it includes both pediatric and adult patients. No other possibility to analyze this sub-cohort due to the division of the ICD-10-related age data into 5-year age ranges could be found. Due to the purely retrospective anonymized data and consequently the lack of traceability of the individual cases of the big data analysis, this group includes a mixed collective of adolescents with possibly still open epiphyseal plates as well as young adults after epiphyseal closure. To avoid a loss of a relevant cohort of patients that could distort the results since this age group was responsible for one of the peaks in age distribution and represents the highest incidence rate, this group was not excluded [40]. Furthermore, a relevant number in our clinical analysis was the teenage age group, but the conclusions drawn from the epidemiological big data analysis of this sub-group were compromised.

Further limitations of the present study certainly exist with respect to the low number of cases and the inhomogeneous patient population with at least three different injury patterns, which require a different approach. As the patients in our clinical analysis were treated by proven specialists in pediatric trauma centers with a high number of cases and expertise, a larger number of patients and uniform treatment concepts could only be achieved implementing a broader, prospective study. In our opinion, randomized studies would only be possible for performing a comparison of conservative versus surgical treatment for the higher-grade types (≥ Rockwood III) of ACJ injuries, in particular the controversial and discussed unstable Rockwood III injuries, in a sensible manner.

## Conclusions

Injuries to the periclavicular joints in childhood and adolescence are rare. Three main categories of most relevant high-grade dislocations have to be distinguished: (1) posterior SCJ dislocation type Allman III, (2) anterior SCJ dislocation type Allman III, and (3) ACJ dislocation type Rockwood III and higher. For posterior SCJ dislocation, it is important to exclude relevant mediastinal concomitant injuries. Clavicular joint injuries tend to affect adolescents rather than children when they are growing and are usually found as mono-injuries. Concomitant injuries are rare in children. The reduction of dislocation is mandatory. Conservatively treated patients show good results. According to our results, primary stabilization with (resorbable) cerclages appears safer. For anterior dislocation, primary stabilization with cerclages is recommended, while in case of relapses or late primary surgery, we recommend an additional concurrent muscle or tendon reconstruction or graft when necessary. While all patients with high-grade ACJ dislocations were treated surgically and achieved restitution ad integrum, data from the literature indicate that conservative treatment can also produce good results in many cases. In unstable ACJ dislocations, our data show none of the surgical methods used to be inferior so far.

It becomes clear that even after our investigation, clear therapy guidelines cannot be established and that it is necessary to have the opportunity to offer various treatment options after an individual decision. Due to the rarity of the clavicular joint injuries in childhood, treatment should be performed by pediatric traumatologists with special experience.
